# Automated multivariate analysis of multi-sensor data submitted online: Real-time environmental monitoring

**DOI:** 10.1371/journal.pone.0189443

**Published:** 2018-01-12

**Authors:** Ingvar Eide, Frank Westad

**Affiliations:** 1 Statoil ASA, Research Centre, Trondheim, Norway; 2 CAMO Software AS, Oslo, Norway; CNRS, FRANCE

## Abstract

A pilot study demonstrating real-time environmental monitoring with automated multivariate analysis of multi-sensor data submitted online has been performed at the cabled LoVe Ocean Observatory located at 258 m depth 20 km off the coast of Lofoten-Vesterålen, Norway. The major purpose was efficient monitoring of many variables simultaneously and early detection of changes and time-trends in the overall response pattern before changes were evident in individual variables. The pilot study was performed with 12 sensors from May 16 to August 31, 2015. The sensors provided data for chlorophyll, turbidity, conductivity, temperature (three sensors), salinity (calculated from temperature and conductivity), biomass at three different depth intervals (5–50, 50–120, 120–250 m), and current speed measured in two directions (east and north) using two sensors covering different depths with overlap. A total of 88 variables were monitored, 78 from the two current speed sensors. The time-resolution varied, thus the data had to be aligned to a common time resolution. After alignment, the data were interpreted using principal component analysis (PCA). Initially, a calibration model was established using data from May 16 to July 31. The data on current speed from two sensors were subject to two separate PCA models and the score vectors from these two models were combined with the other 10 variables in a multi-block PCA model. The observations from August were projected on the calibration model consecutively one at a time and the result was visualized in a score plot. Automated PCA of multi-sensor data submitted online is illustrated with an attached time-lapse video covering the relative short time period used in the pilot study. Methods for statistical validation, and warning and alarm limits are described. Redundant sensors enable sensor diagnostics and quality assurance. In a future perspective, the concept may be used in integrated environmental monitoring.

## Introduction

Sensor based environmental monitoring has during the past years been used in long-term monitoring programs and provides the possibility of online submission of data more or less continuously [1–4]. This provides large amounts of data and challenges associated with submission, interpretation and storage [5]. Each sensor variable may be monitored individually, however, an alternative is to monitor all variables simultaneously using multivariate data analysis. We hypothesize that this represents an efficient interpretation of large amounts of data submitted frequently and is also more sensitive in terms of early detection of systematic changes and time-trends in the overall response pattern before changes are evident in individual variables.

Challenges with sensor based environmental monitoring are associated with long-term reliability and resistance to bio-fouling of sensors and subsea systems [2]. This requires measures to distinguish changes in the environment from shift in sensor response (sensor diagnostics and quality assurance).

Multivariate data analysis has successfully been used in several marine environmental studies for the interpretation of large data sets, for example on heavy metals and biomarker response [6], on spatial and temporal distribution of heavy metals in seawater and sediments [7], on element release from sediments [8], on Polycyclic Aromatic Hydrocarbons (PAH) in marine and freshwater sediments [9–10], and to predict toxicity from sediment chemistry [11]. In a recent study, multivariate data analysis was used in integrated environmental monitoring, comprising not only chemical, physical and biological variables but also discharge data at the Peregrino oil field off the coast of Brazil [12].

Several studies have been published where various strategies for decomposing time series data have been applied [13–15]. Frequently applied methods are analysis of power spectra by means of Fast Fourier Transform or the Hilbert transform, Empirical Mode Decomposition, wavelets and Single Spectral Analysis based on autocorrelation.

The present paper describes a pilot study using automated multivariate data analysis of multi-sensor data submitted frequently or continuously in real-time. The major purpose is efficient monitoring of many variables simultaneously and early detection of changes and time-trends in the overall response pattern before changes were evident in individual variables. So far, the concept has been applied on the cabled LoVe Ocean Observatory located at the seabed off the coast of Lofoten-Vesterålen, Northern Norway [3]. LoVe is an acronym for Lofoten-Vesterålen. Data from multiple sensors (a total of 88 variables) are recorded in real-time. The present paper describes how these data are analysed consecutively using automated Principal Component Analysis (PCA) and how the results are interpreted and visualized. Furthermore, methods for statistical validation, the use of warning and alarm limits, and the use of redundant sensors for sensor diagnostics and quality assurance are described.

## Materials and methods

### Lander and sensors

The LoVe Ocean Observatory consists of a lander positioned at 258 m depth 20 km off the coast of Lofoten-Vesterålen, Northern Norway [3]. The location was chosen because it is strategic in relation to a major current into the Barents Sea. The area hosts coral communities, and is important for cod spawning. The lander is equipped with different sensors, each providing single or multiple outputs.

Current speed is measured every 10 min, using a short-range Nortek Aquadopp Profiler ADCP (600 kHz) and a long-range Nortek Continental ADCP (193.5 kHz) both from Nortek AS, 1351 Rud, Norway. Due to weakening of the signal-to-noise ratio as a function of the distance to the sea floor the Aquadopp Profiler was used to obtain data from 3 to 23 m above seabed with 2.5 m intervals (9 depths) and the Continental sensor recorded signals for every 5 m from 8 to 153 m above seabed (30 depths). Both sensors recorded current speed in two directions (E and N) with overlap at 8, 13, 18 and 23 m above seabed.

Temperature, conductivity, and turbidity were measured every five minutes. An AADI 4319 (Aanderaa Data Instruments AS, Bergen, Norway) was used for conductivity and temperature measurements. Also, the two sensors for current speed had temperature sensors which gives redundancy and the possibility to detect if one or more of the sensors fail. Salinity was calculated from conductivity and temperature [16] and are not independent observations as such. The turbidity measurements were performed with an optical sensor (AADI 4112) that measured scattered light at 880 nm. Chlorophyll was measured every hour at 470 nm using a Seapoint chlorophyll fluorometer (Seapoint Sensors Inc., Exeter, NH 03833, USA).

Acoustic data were provided by an echosounder (Simrad EK60 70 kHz Splitbeam) with two 70 kHz Simrad ES70-CD pressure resistance transducers (in vertical and horizontal directions). Biomass index (volume backscattering strength, Sv) at three different depth intervals (5–50, 50–120, 120–250 m) was calculated from the acoustic data [17–18].

Further details on the observatory, lander and sensors are available on the LoVe web site, http://love.statoil.com/

### Data

The LoVe Ocean Observatory is cabled and data are submitted online from each sensor, however, time resolution varies. Data alignment was used to arrange a table with all variables on a common time scale with one hour intervals. Linear interpolation was chosen as alignment method, as the sampling rate was rather high compared to the expected changes in the conditions due to natural and seasonal variations. The data used in the present pilot study were from the period May 16 to August 31, 2015 with all 12 sensors submitting data with a few interruptions resulting in a total of 1823 observations after alignment, each observation containing values for 88 variables (S1 Table). There were only 24 missing values in the dataset used in the present study, implying that they will not affect the model in a significant way. However, the NIPALS algorithm implemented in the software handles missing values directly. Furthermore, the software gives an alert if one or more variables has more than 10% missing values.

Missing data are associated with the major maintenance problem so far namely the robustness of the underwater hardware and software. This is first of all registered as missing data.

Since the data used in the present paper necessarily are historic, the automated real-time PCA model is illustrated with score and loading plots, plots of raw data and a time-lapse video.

### Multivariate data analysis

Principal component analysis, PCA [19] was used as an exploratory method to evaluate similarities and differences between observations and for interpreting the relationships between the variables. The results from PCA are visualized in score and loading plots. Each observation comprising all variables at the same time becomes one point in the score plot. Each variable is represented by one point in the loading plot. The loadings represent the underlying dimensions and how important the individual variables are in describing these. Thus, the loadings define the model space in terms of all variables on which new observations can be projected. Simultaneous interpretation of score and loading plots reveal the important variables and corresponding observations.

Many methods are available for analyzing one data table, e.g. the traditional Factor Analysis, Independent Component Analysis, PCA followed by Varimax rotation or oblique rotations, Multidimensional scaling etc. Time series analysis in both univariate and multivariate versions would have been a complimentary approach. References [13–15] describe methods based on transforming the time series into frequency domain, applying autocorrelation to generate lagged data suited for PCA e.g. As the time period was limited, no time series models were included. Alternatively, with the multi-sensor data, one could envision Fourier Transforms on the score vectors from PCA. However, this will not yield the same possibility for on-line monitoring for the individual observations. Furthermore, PCA gives PCs that reflects the background information inherent in the sensors and the environmental monitoring.

Initially a calibration model was established for a defined period. New observations were projected onto the calibration model and visualized in a score plot and other plots for detecting trends and outliers. With the data available for the present paper, the calibration model was based on the period May 16 to July 31, 2015 (1311 observations). The new observations to be projected on the calibration model were from August 1–31, 2015 (512 observations). The calibration data had occasionally missing values which in the software was automatically handled by omitting these values in estimation of the model variables.

**Fig 1:**
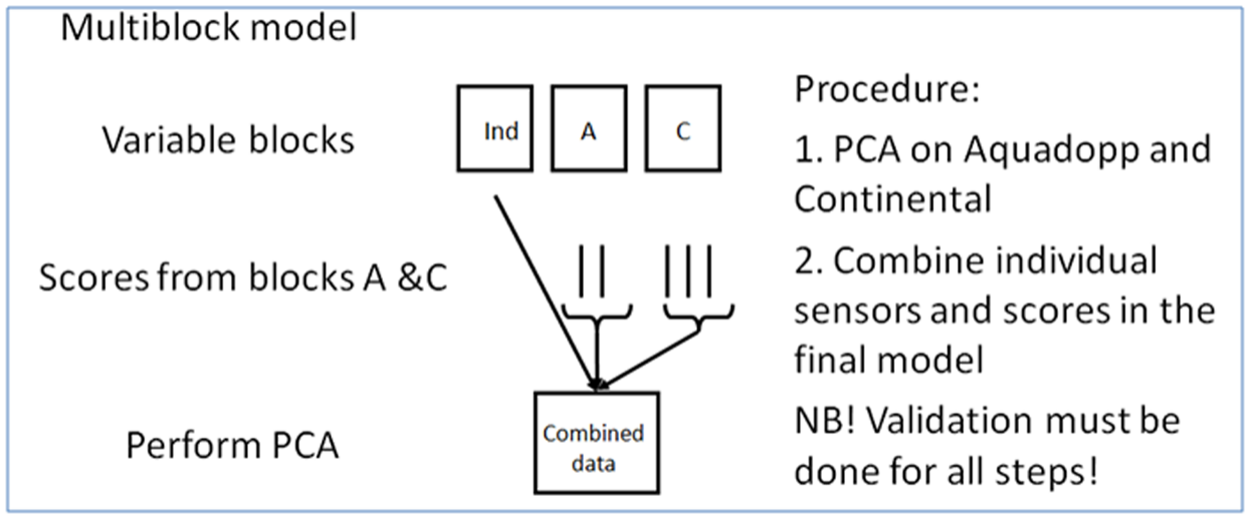
Illustration of the multi-block model. Ind: variable block for individual sensors; A: variable block for Aquadopp profiler; C: variable block for Continental ADCP.

The calibration model was based on 10 individual sensors and the Aquadopp and Continental sensors which provided current speed in two directions at 9 and 30 different depths, altogether 18 and 60 individual variables, respectively. However, the 78 variables from the two current speed sensors would dominate the other 10 individual sensor variables and would also give an "overcrowded" loading plot. Therefore, the two sets of data on current speed were subject to two separate PCA models and the score vectors from these two models where combined with the 10 individual sensors in a multi-block PCA model. An illustration of the multi-block model is shown in Fig 1. All variables were mean centred and scaled to unit variance so that they have the same possibility to influence the model regardless of the original unit and their variance. The abbreviations used for some variables in the score and correlation loading plots are shown in Table 1.

**Table 1:** Abbreviations used for some variables in the correlation loading plot (Fig 2) and the contribution plots (Figs 4 and 6).

Abbreviation	Variable name
Biomass 5m	Biomass 5–50 m
Biomass 50m	Biomass 50–120 m
Biomass 120m	Biomass 120–250 m
TConti	Temperature at Continental ADCP
TAqua	Temperature at Aquadopp profiler
TCond	Temperature at Conductivity sensor
C_PC1	PC1 Continental ADCP
C_PC2	PC2 Continental ADCP
C_PC3	PC3 Continental ADCP
A_PC1	PC1 Aquadopp profiler
A_PC2	PC2 Aquadopp profiler
A_PC3	PC3 Aquadopp profiler

The models were validated with respect to explained and validated variance and by interpretation of the correlation loadings and score plots to assess the correct dimensionality of the models [20]. Four components were found to be the optimal number. Once the number of PCs were determined, critical statistical limits for detecting outliers were estimated as described in the paragraphs below. In addition, redundant sensors were used for sensor diagnostics and quality assurance.

The critical limit for samples to lie inside the model space was found from the Hotelling's *T*^2^ statistics [19,21] which is a multivariate extension of the t-test statistics. It is known that univariate Statistical Process Control (SPC) does not maintain the significance level in terms of t-test or confidence interval, as more individual variables are included, reducing the ability to detect out-of-control situations [19,21]. The Hotelling's *T*^2^ statistics is a measure of how extreme the observation is in the model space and is the distance to the model centre as spanned by the principal components. Thus, it can be used to track systematic changes in the environment and detect situations where a system is outside normal conditions. The Hotelling's *T*^2^ critical limit is shown as a confidence ellipse in the 2-dimensional score plot or as a line plot with an upper critical limit. The Hotelling's *T*^2^ value for individual samples can be broken down into the individual variables, their so-called contributions.

The Q-residual is the sum of squares of the residuals over the variables for each observation [19,21]. They may be used to detect statistical outliers where the pattern for the variables for one or more observations do not match the model. A high Q-residual will occur if a new observation has a different value e.g. if one of redundant sensors deviates from the correlation as found in the model. Similar to the Hotelling's *T*^2^, the contribution from individual variables to the sample Q-residual can be displayed for diagnostic purposes.

The software used for developing the model was the Unscrambler X 10.5 (CAMO Software, Oslo, Norway) and the model together with the data sources were configured in Process Pulse II for real-time monitoring and also for making the time-lapse video simulating real-time monitoring. The Process Pulse II software allows various data sources (sensors) to be used for classification by means of projection onto PCA models with critical limits for outlier detection.

## Results and discussion

### Calibration and online projection

**Fig 2:**
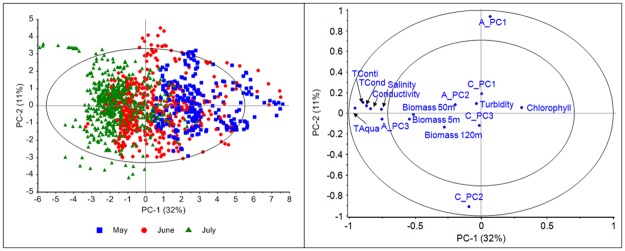
Score plot (left) and correlation loading plot (right) for the calibration model.

Fig 2 shows score and correlation loading plots for the calibration model based on data from May 16 to July 31. The score plot (left) shows clearly three groups of observations (different colours for each month) although there is overlap due to a gradual change from one month to another. The outer circle in the correlation loading plot represents 100% explained variance and the inner circle 50% explained variance for the individual variables. The plot reveals that the three temperature sensors are highly inter-correlated and describes PC1 together with conductivity, salinity and the 3rd score from the Aquadopp model. As can be seen by colouring according to month, the temperature increases from May to July in a systematic way. PC2 is mainly spanned by two underlying variations in the current speed data, PC1 from the Aquadopp model and PC2 from the Continental model. The detailed interpretation showed that this variation describes increased speed as a function of the distance to the sea bed, and more pronounced in the eastern direction. The opposite position of the two variables is due to opposite directions in the individual PCA models for Aquadopp and Continental themselves. However, this not does affect the interpretation as such.

The three Biomass variables are mostly described by PC2 (11% explained) and PC3 (8%). The fourth PC (8%) reflects variation in the first PC from the Continental sensor, which in the corresponding loading plot shows to be current speed in the east-west direction from 100–150 meters above the sea level (not shown). As can be seen in Fig 2 (right), turbidity is not well explained in the calibration model implying low and unsystematic variation.

The attached time-lapse video (S1 Video) illustrates the concept of real-time monitoring and the projections of the new observations on the calibration model in a score plot. Each grey point represents one observation in the calibration model. Once the video starts, new points are added to the score plot every second outside (red points) or inside (green points) the Hotelling’s *T*^*2*^ ellipse (one month monitoring compressed to 8 min, however, only last 2 min shown). Also, raw data for two temperature sensors are shown in the video. At the LoVe web site also plots with raw data, contribution, Q-residuals, etc. will be available for all new observations Particularly important is identification of the variables responsible for observations located outside the Hotelling’s *T*^*2*^ ellipse, alternatively outside another defined warning or alarm limit. Note that the tests for outliers is based on the model with four PCs, thus the Hotelling's *T*^*2*^ ellipse in Fig 2 reflects only the first two PCs. Nevertheless, these two first PCs are the ones with the highest contribution to the critical value for the Hotelling's *T*^*2*^.

**Fig 3:**
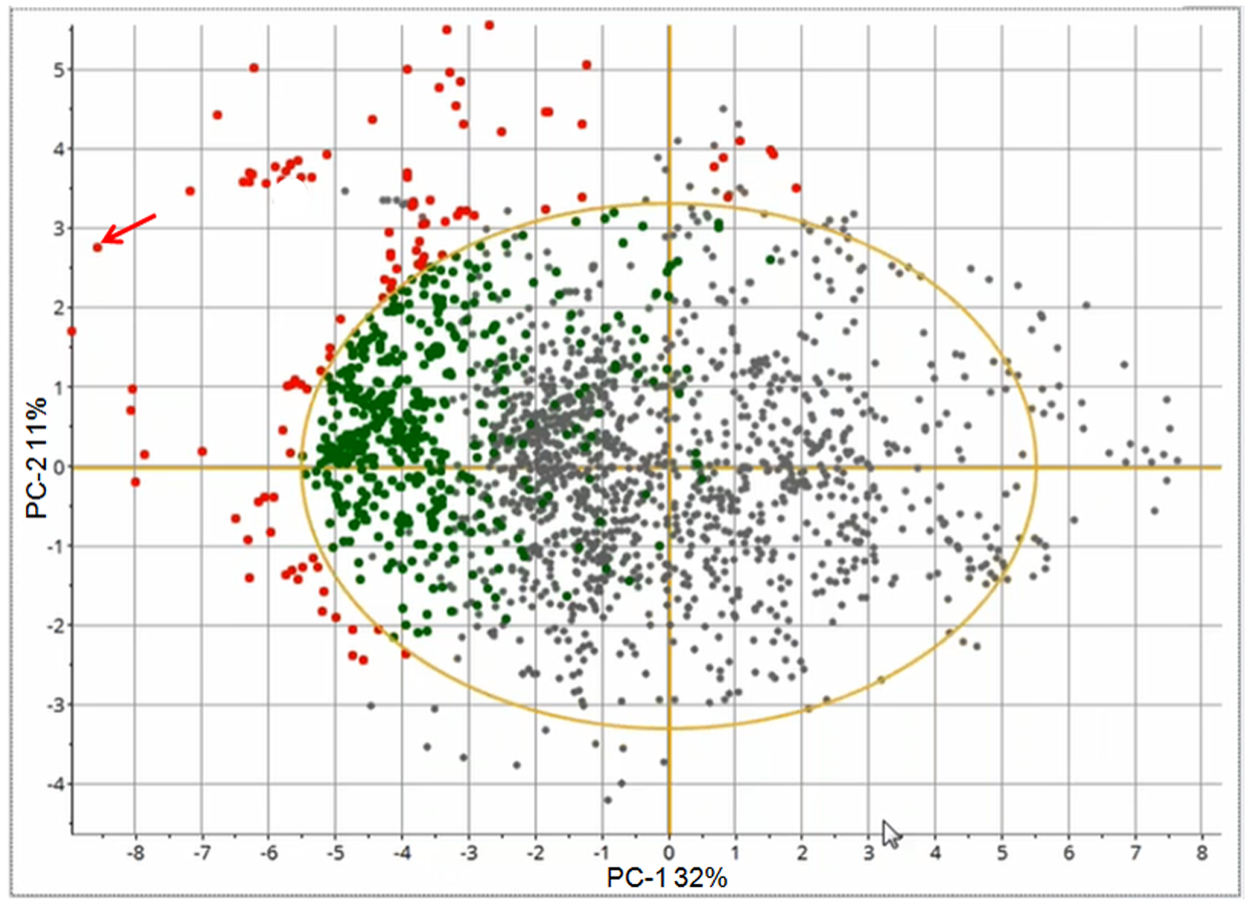
Score plot with projection of the August data on the calibration model.

**Fig 4:**
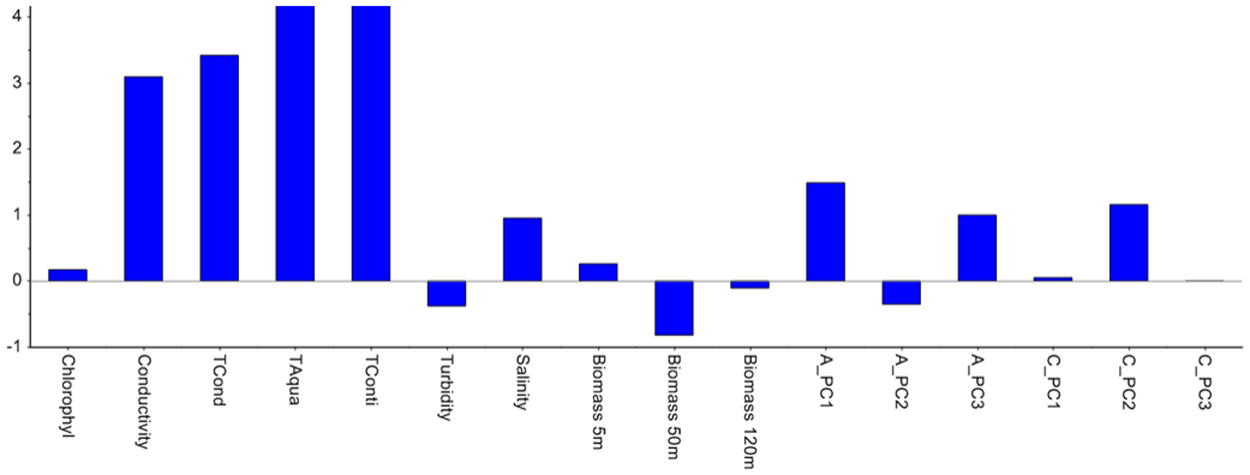
Hotelling's *T*^2^ contribution plot representing one observation (marked with an arrow in Fig 3).

**Fig 5:**
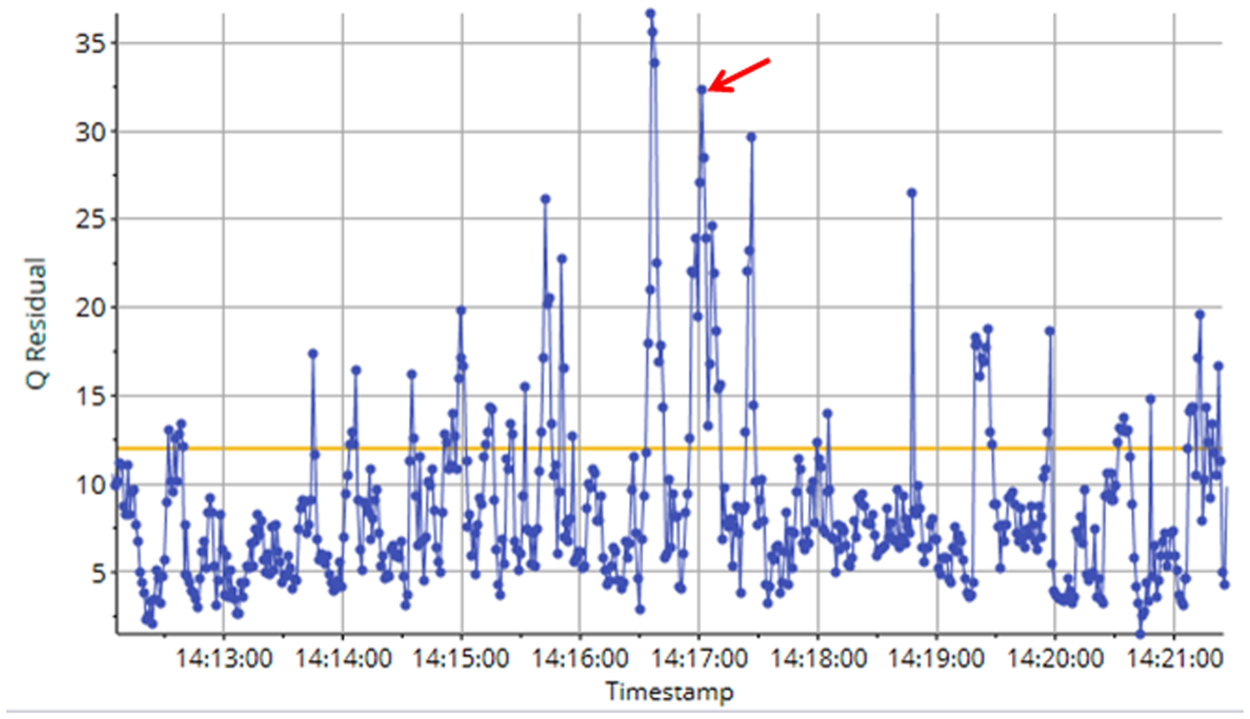
Q-residual plot for all August observations.

**Fig 6:**
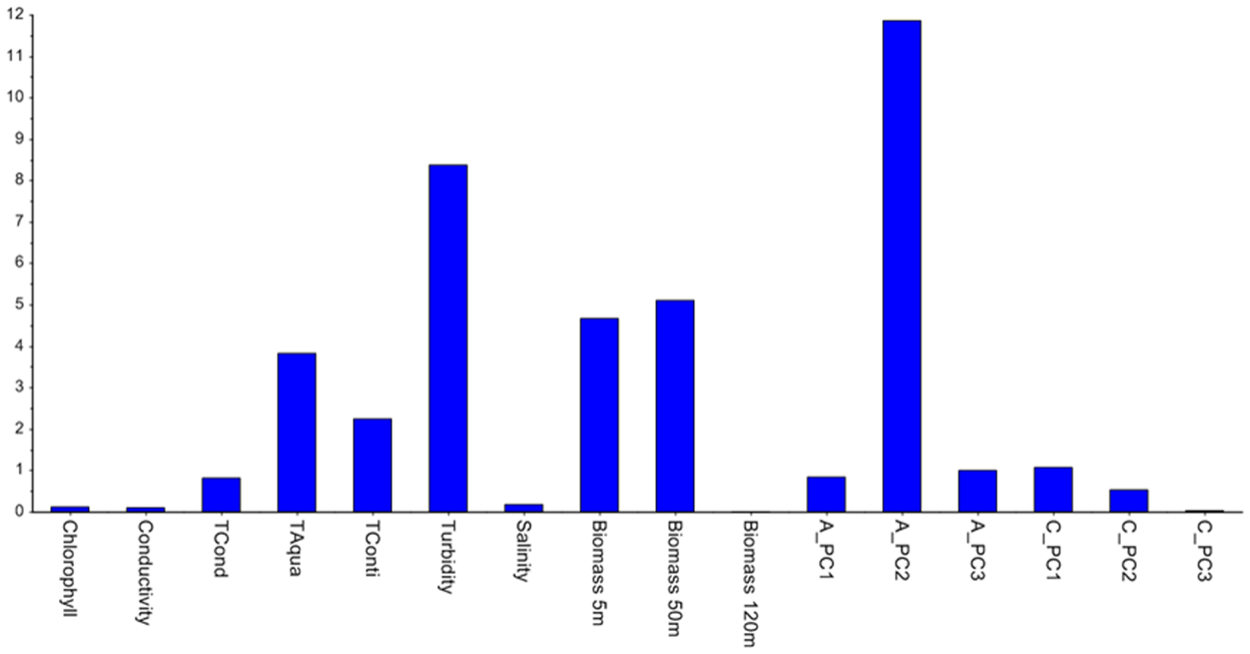
Q-residual plot for one August observation (marked with an arrow in Fig 5).

Fig 3 shows the score plot after the very last August observation has been projected (the end of the time-lapse video). As the new observations from August are located to the left in the score plot, and the temperature variables are located to the left in the correlation loading plot, it means that the temperature is increasing in August, as expected and confirmed by the Hotelling’s *T*^*2*^ contribution plot in Fig 4. Thus, the systematic trend towards higher temperature continues. As this study only involves data from four months, no full-year seasonal variability can be interpreted, but a yearly cycle would have been expected. This is also a reason why time-series analysis which were used in references [13–15] was not pursued. The contribution plot provides identification of the variables that have changed in one observation (marked with an arrow) in the score plot in Fig 3. Fig 5 shows the Q-residual plot for all August observations and Fig 6 shows the Q-residual contribution plot for one August observation marked with an arrow in Fig 5. These two plots illustrate the ability to find anomalies of individual variables interactively with a few mouse-clicks, and thus there is no need to monitor all individual variables in line plots on a time scale. Nevertheless, in this application, PCA is in essence used as a classification method where there is only one "class", namely the model for three months, and any anomaly will be detected by means of the statistical limits and drill-down plots.

Turbidity was located close to origo in the correlation loading plot for the calibration model (Fig 2) implying low explained variation and high residuals, and poor correlation with other variables. When projecting the august data onto the model, the Q-residual for turbidity was high (Fig 6). This led to inspection of the raw data which revealed that the values peaked at approximately125 for most of the observations. Turbidity peaked because the sensor was not properly calibrated and the range was set too low. It is obviously critical that the sensors are properly calibrated prior to deployment so that the PCA can detect possible changes. The maintenance protocol will be improved to cover satisfactory calibration of sensors prior to deployment and in addition recalibration to quantify possible deviations after retrieval from seabed.

Since explained variation was low and unsystematic and correlation with other variables were low, turbidity had low influence on the model.

Fig 3 and the time-lapse video illustrate that the observations from August are different from the preceding months. This was of course expected. However, the data serve to illustrate the basic principles of the online automated PCA in this pilot study. The present work was a pilot study with data from approximately four months only. In a future perspective, longer periods will be used in environmental monitoring. However, due to seasonal variations it may be necessary to have different calibration models for different seasons or a stepwise recalibration.

### Redundant sensors and QA

The online PCA detects changes and obviously requires calibrated sensors and a period with correct signals in order to make a calibration model. Distinguishing between wrong data and trend changes can be done with redundant sensors, and by identifying variables with low explained variation and high residuals in observations outside a critical limit.

As described earlier, low explained variation and high residuals were the reason for the inspection of raw data for turbidity revealing that the turbidity sensor peaked providing essentially the same value. If another turbidity sensor had been installed, it would be possible to detect a change in one of them. At the time of installation, the system did not have the possibility to screen individual variables based on pre-set lower and upper limits. Thus, the detection of anomalies was by means of the PCA and drill-down into specific plots as described above. Nevertheless, screening individual values to filter out obvious sensor failure and anomalies would make the monitoring more user friendly for the operators.

**Fig 7:**
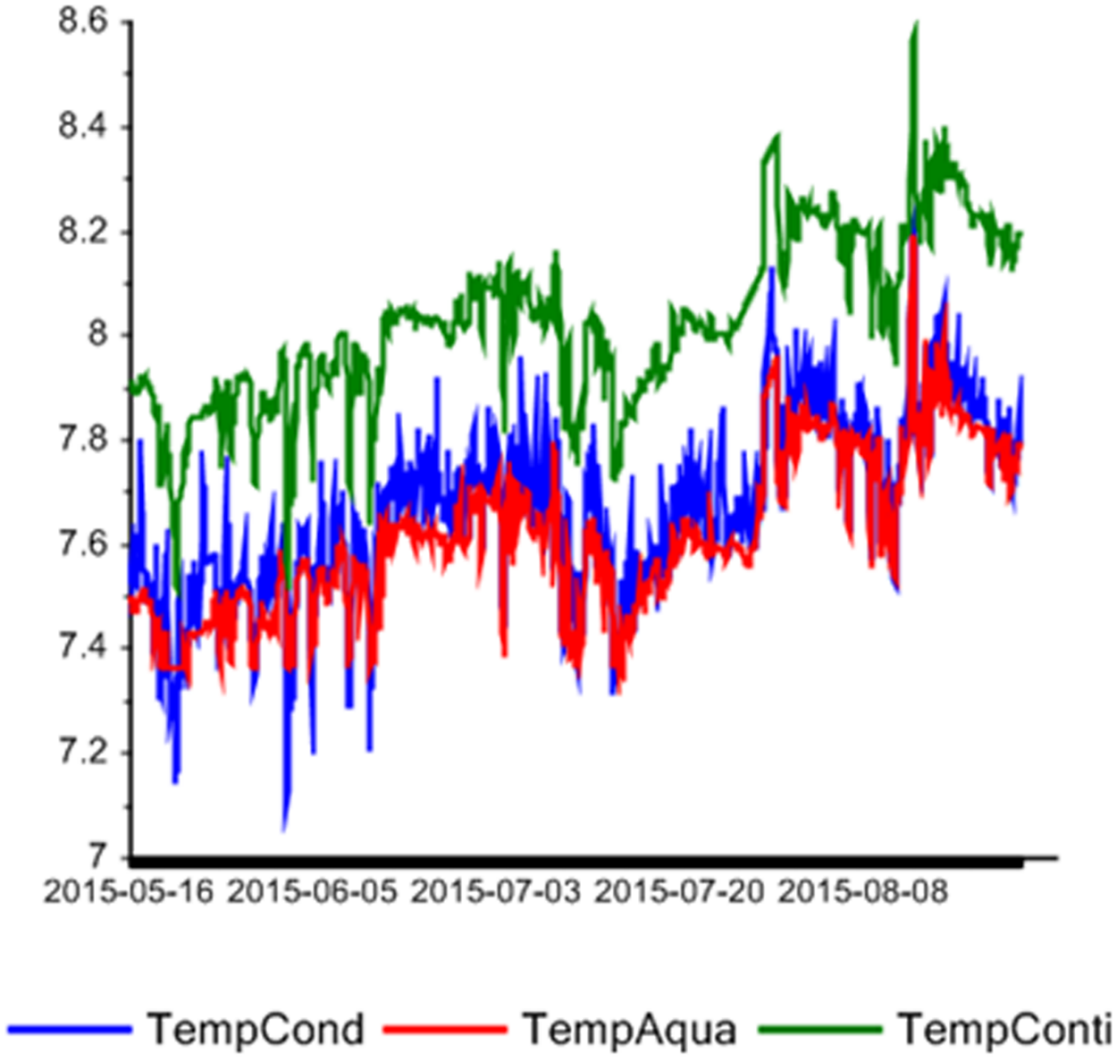
Line plot of the three temperature sensors.

There were three temperature sensors, thus the system has redundancy with respect to temperature. Furthermore, both sensors for current speed measured with overlap at depths 8–23 m above sea bed. One of the advantages with the multivariate methods is that if the correlation between some sensors is changing this will be detected by the outlier statistics. Fig 7 shows a line plot of the three temperature sensors for the time period May 16 to August 31. As can be seen there is an offset between them but the correlation is high.

**Fig 8:**
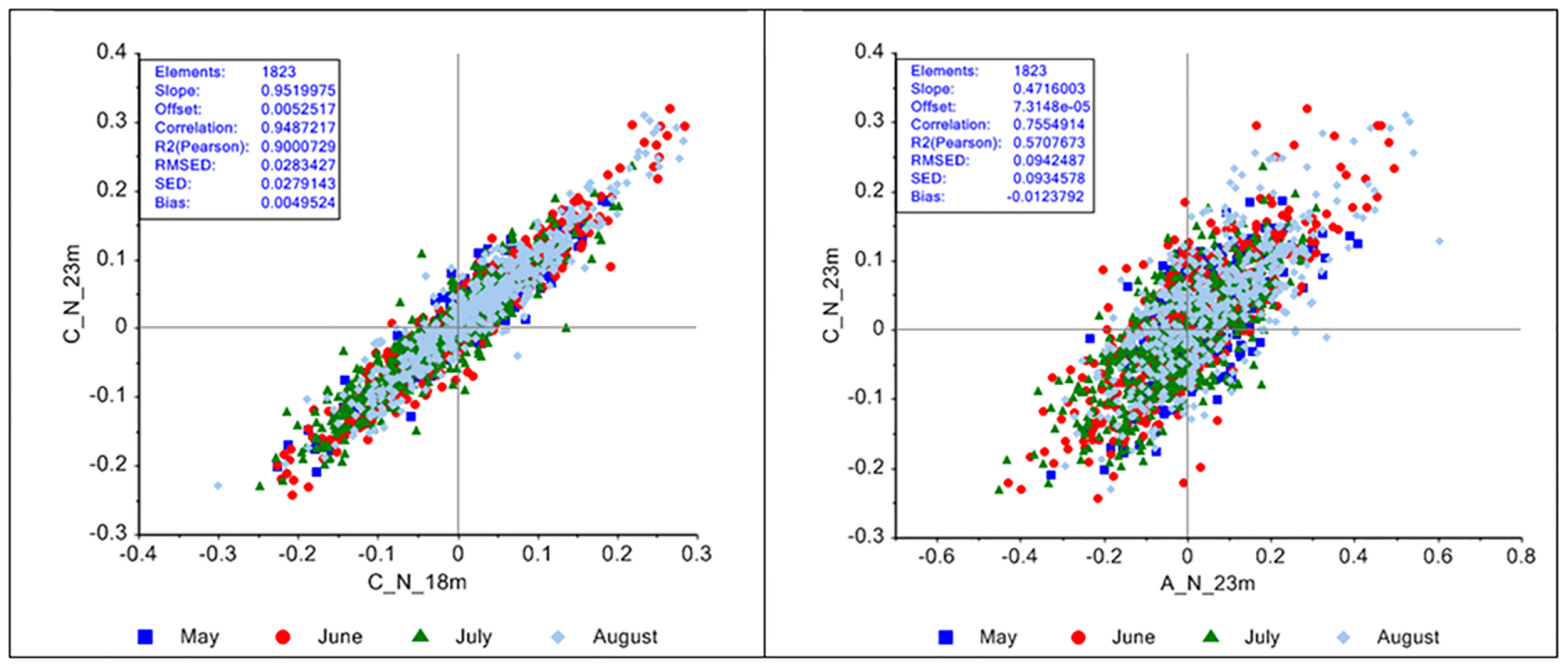
Scatter plot of the current speed north at 18 and 23 m above sea bed for Continental (left) and at 23 m above sea bed for Continental and Aquadopp (right). A: Aquadopp; C: Continental; N: north.

The correlation loading plot in Fig 2 shows correlation between PC1 for Aquadopp and PC2 for Continental (correlation is 0.80). The explained variance for the Aquadopp model was 68, 19 and 6% respectively for the three PCs. For the Continental sensors the values were 67, 15 and 5%. Fig 8 (left) shows a scatter plot of the raw data for the Continental sensor in the north direction measured at 18 and 23 m above sea bed, respectively. As can be seen the correlation is 0.95, thus there is a linear dependency between currents at various depths. This illustrates redundancy between variables within one sensor system. Furthermore, as both sensors for current speed measured with overlap between 8 and 23 m above sea bed, they also provide redundancy in the system. Fig 8 (right) shows the correlation (0.76) between the northern currents at 23 m for Aquadopp and Continental. The corresponding correlation between the eastern currents at 23 m for Aquadopp and Continental is 0.86 (scatter plot not shown).

**Fig 9:**
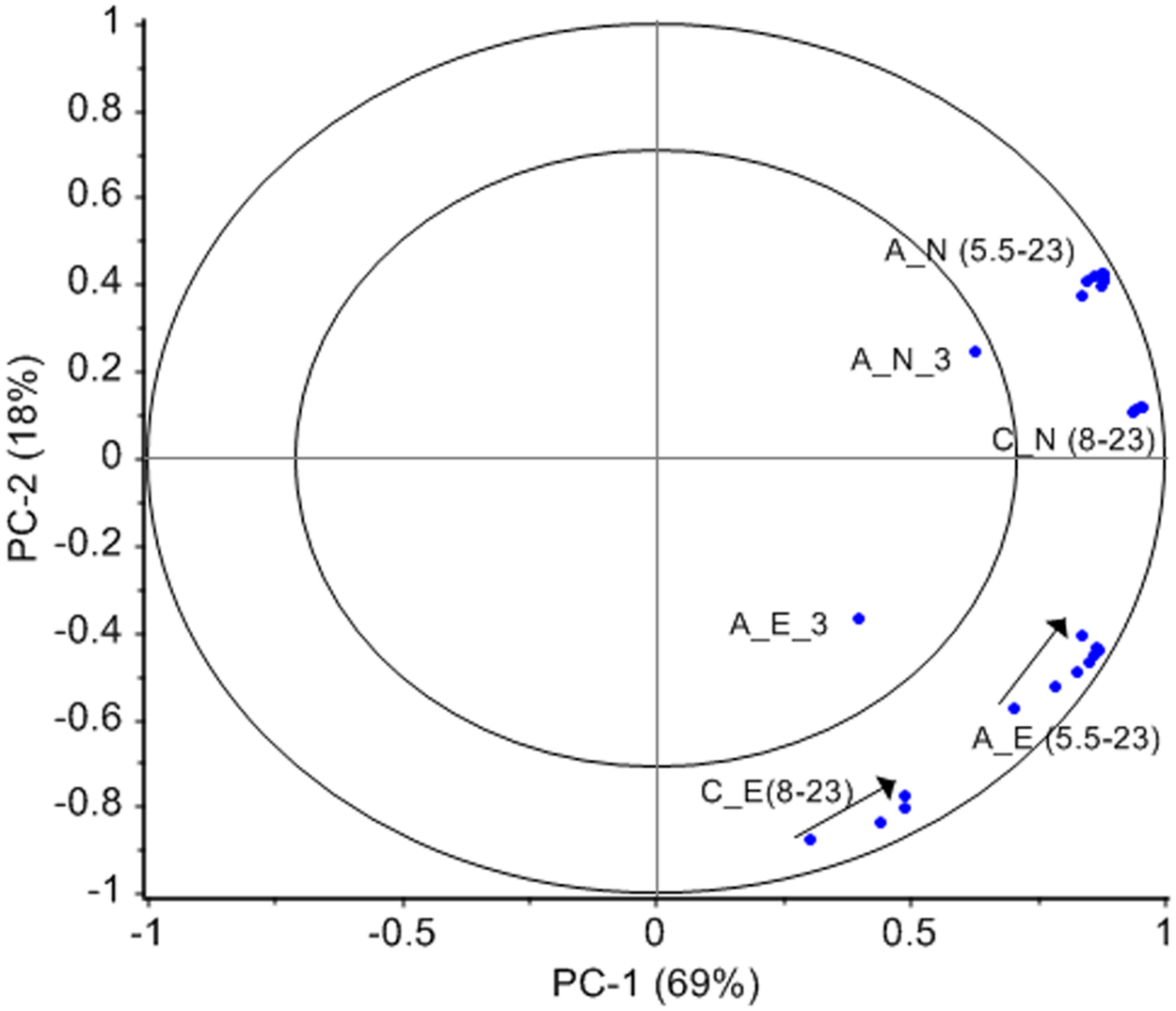
Correlation loading plot after PCA on current speed data 3–23 m above sea bed from both current speed sensors. A: Aquadopp; C: Continental; E: east; N: north.

A general visualization of the correlation between the various depths can be shown in the correlation loading plot for a combined model for the two sensors using data from 3–23 m above seabed. Fig 9 shows that there exists a high correlation for these depths except at 3 m (which was only measured with Aquadopp). By interpretation of PC3, it was found that the speed at 3 m mainly showed another systematic variation over time which can be explained by the proximity to the sea floor. There is also a systematic difference between the two sensors which would not have been evident from looking at a direct 2D correlation table. The statistics in Fig 8, however, shows that the bias is close to zero, so there is not a difference due to offset as in the case with the temperature sensors.

Fig 9 shows a systematic gradient in eastern current from 8–23 m for the Continental and from 5.5–23 m for the Aquadopp profiler. This gradient is not seen in northern currents. Current speed (N and E) at 3 m was only measured by the Aquadopp profiler and the two variables have separate positions in the correlation loading plot.

The correlation loading plot in Fig 9 capture much of the information inherent in the two individual PCA models for the Continental (8–153 m) and the Aquadopp (3–23 m) profiler which provided the score vectors used in the multi-block model (Fig 2). The PC1 from the Aquadopp model describes a systematic gradient from 3 to 23 m above sea floor; the signal stabilizes at 8 m. This systematic variance is more pronounced in the eastern direction. PC2 from the Continental model shows a similar structure from 8 to 153 m being more pronounced in the eastern direction.

## Concluding remarks

The use of multivariate data analysis and the multi-block approach has been most useful in obtaining the important correlations and systematic trends in the large data set with many variables and observations. The LoVe Ocean Observatory represents the first application of online automated multivariate data analysis in environmental monitoring. At LoVe the application is used for baseline studies to reveal natural and seasonal variation, time trends, and also to detect certain events in addition to sensor failure. In this pilot study, the time period was relatively short with three months for calibration and one month for projections. In a future perspective, the purpose is integrated environmental monitoring and early preventive measures in areas with discharges. In addition to discharge data also data from spectra, images and biosensors may be incorporated in the monitoring program. In fact, the benefit of the multivariate data analysis becomes more evident the more variables that are incorporated in the monitoring. Furthermore, the advantage with the online automated multivariate data analysis is the detection of changes in many variables simultaneously in real-time before they are evident in individual variables. Hence, it should become an important tool for decision making in integrated environmental monitoring.

## Supporting information

S1 Table All data with 1823 observations and 88 variables. (XLSX)

S1 Video Time-lapse video showing projection of August observations on the calibration model and raw data for two temperature sensors. One month monitoring compressed to 8 min, however, only last 2 min shown.
(MP4)
